# Bioinformatics for cancer immunology and immunotherapy

**DOI:** 10.1007/s00262-012-1354-x

**Published:** 2012-09-18

**Authors:** Pornpimol Charoentong, Mihaela Angelova, Mirjana Efremova, Ralf Gallasch, Hubert Hackl, Jerome Galon, Zlatko Trajanoski

**Affiliations:** 1 Biocenter, Division of Bioinformatics, Innsbruck Medical University, Innrain 80, 6020 Innsbruck, Austria; 2 INSERM U872, Integrative Cancer Immunology Laboratory, Paris, France

**Keywords:** Databases, Epitope prediction, Next-generation sequencing, Mathematical modeling, Bioinformatics, Immunotherapy

## Abstract

Recent mechanistic insights obtained from preclinical studies and the approval of the first immunotherapies has motivated increasing number of academic investigators and pharmaceutical/biotech companies to further elucidate the role of immunity in tumor pathogenesis and to reconsider the role of immunotherapy. Additionally, technological advances (e.g., next-generation sequencing) are providing unprecedented opportunities to draw a comprehensive picture of the tumor genomics landscape and ultimately enable individualized treatment. However, the increasing complexity of the generated data and the plethora of bioinformatics methods and tools pose considerable challenges to both tumor immunologists and clinical oncologists. In this review, we describe current concepts and future challenges for the management and analysis of data for cancer immunology and immunotherapy. We first highlight publicly available databases with specific focus on cancer immunology including databases for somatic mutations and epitope databases. We then give an overview of the bioinformatics methods for the analysis of next-generation sequencing data (whole-genome and exome sequencing), epitope prediction tools as well as methods for integrative data analysis and network modeling. Mathematical models are powerful tools that can predict and explain important patterns in the genetic and clinical progression of cancer. Therefore, a survey of mathematical models for tumor evolution and tumor–immune cell interaction is included. Finally, we discuss future challenges for individualized immunotherapy and suggest how a combined computational/experimental approaches can lead to new insights into the molecular mechanisms of cancer, improved diagnosis, and prognosis of the disease and pinpoint novel therapeutic targets.

## Introduction

Recent mechanistic insights obtained from preclinical studies and the approval of the first immunotherapies have motivated increasing number of academic investigators and pharmaceutical/biotech companies to further elucidate the role of immunity in tumor pathogenesis and to reconsider the role of immunotherapy. Several factors contributed considerably to this renaissance phase of cancer immunology and immunotherapy [1].

First, major advances in immunology over the past 30 years improved our understanding of the complex interaction between the immune system and the tumor [2]. The immune system can respond to cancer cells by reacting against tumor-specific antigens or against tumor-associated antigens. The antigenic determinants, epitopes, are presented on the cell surface, where they can be recognized by T cells or antibodies, eventually eliciting tumor destruction or enforcing proliferation. Cancer immunosurveillance is considered to be an important host protection process to inhibit carcinogenesis and to maintain cellular homeostasis [3]. Extensive work in experimental systems has elucidated some of the mechanisms underlying spontaneous antitumor immunity and has formed the basis for the cancer immunoediting hypothesis. This hypothesis divides the immune response to cancer into the “three E’s” which are elimination, equilibrium, and escape [4–6].

Second, there is increasing clinical evidence that the immune system influences the recurrence of cancer. For example, our previous results have shown the close correlation between the “high” intra- and peri-tumoral adaptive immune reaction in colorectal carcinoma and a good prognosis, and inversely, a “low” density of T cells was correlated with a poor prognosis [7, 8]. In fact, of all the various clinical and histopathologic criteria currently available, the immune T cell infiltrate was shown to be the most important predictive criteria for survival [7–9].

Third, FDA approval of two cancer immunotherapies: (1) ipilimumab antibody directed against CTLA-4, a molecule that downregulates T cell activation for the treatment of melanoma, and (2) sipuleucel-T, a therapy consisting of autologous PBMC activated with the prostatic acid phosphatase; prostate cancer–associated antigen fused to GM-CSF for the treatment of patients with advanced hormone-refractory prostate cancer. Over and above, recent promising results for the blockade of programmed death 1 (PD-1), an inhibitory receptor expressed by T cells [10, 11], are likely to provide a new benchmark for antitumor activity in immunotherapy and will initiate a number of studies for future multimodal therapy. Historically, the treatment methods for the different types of cancers were surgery, radiation therapy, chemotherapy, or combinations of these to limit the progression of malignant disease. The fourth modality of immunotherapy is now starting to be used in clinical practice and will become a standard treatment for a variety of cancers [2, 12].

Fourth, recent technological advances [e.g., next-generation sequencing (NGS)] are providing unprecedented opportunities to draw a comprehensive picture of the tumor genomics landscape and ultimately enable individualized treatment. Due to the rapid declination of costs per base pair, NGS projects are now affordable even for small- to mid-sized laboratories. Point mutations, chromosomal rearrangements, translation from cryptic start sites or alternative reading frames, splicing aberrations, and over-expression have all been reported as sources of tumor antigens [3, 13, 14] and can be now readily detected. It is noteworthy that recent study showed a proof-of-concept in which somatic mutations are first detected using NGS, then the immunogenicity of these mutations is defined, and finally, mutations are tested for their capability to elicit T cell immunogenicity [15]. Thus, tailored vaccine concepts based on the genome-wide discovery of cancer-specific mutations and individualized therapy seem technically feasible.

However, the increasing complexity of the generated data and the plethora of bioinformatics methods and tools for the analysis pose considerable challenges. In this review, we describe current concepts and future challenges for the management and analysis of data for cancer immunology and immunotherapy. We first highlight publicly available databases with specific focus on cancer immunology including databases for somatic mutations and epitope databases. We then give an overview of the bioinformatics methods for the analysis of next-generation sequencing data (whole-genome and exome sequencing) as well as bioinformatics tools for epitope prediction, integrative data analysis, and network modeling. Mathematical models are powerful tools that can predict and explain important patterns in the genetic and clinical progression of cancer. Therefore, a survey of mathematical models for tumor evolution and tumor–immune cell interaction is included. Finally, we discuss future challenges for individualized immunotherapy and suggest how a combined computational/experimental approaches can lead to new insights into the molecular mechanisms of cancer, improved diagnosis, and prognosis of the disease and pinpoint novel therapeutic targets.

## Data sources

**Fig. 1:**
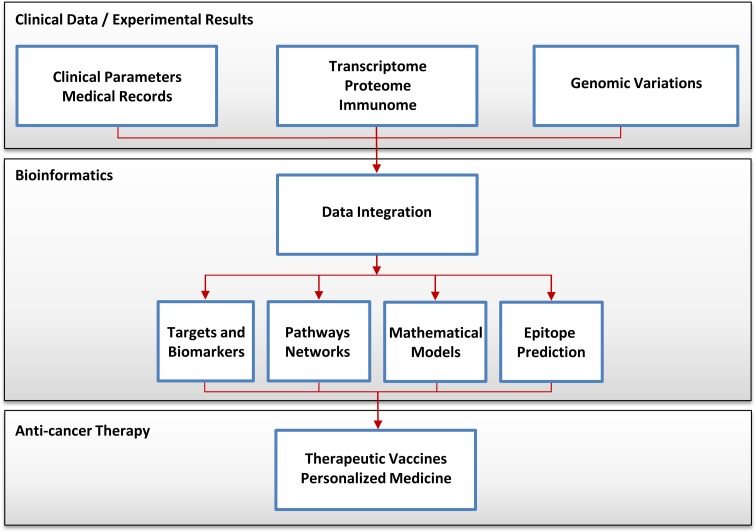
Data and information flow in cancer immunology research. The datasets are integrated from clinical observations, medical records, “omic” technologies, and the next-generation sequencing technology and analyzed by using bioinformatics methods. Cancer researchers are using these data to extract information for diagnosis, classification, prognosis, and therapeutic guidance. Furthermore, the multi-parametric data can lead to the improvement of the immunotherapy and can be exploited for patients benefit using individualized therapeutic cancer vaccines

The continuous improvement of existing technologies for large-scale data generation like microarrays and proteomics, as well as the development of novel powerful technologies including NGS and high-content techniques, led to an increased use in cancer research. Figure 1 illustrates the data and information flow in contemporary cancer immunology research and, in near future, also in personalized cancer immunotherapy. Without surprise, within the last few years, the amount of data generated and deposited in publicly available databases exploded. Thus, a cancer researcher can address today a specific question and not only by generating proprietary high-throughput data but also by accessing and mining available datasets. We therefore describe cancer databases and databases for cancer immunology.

### Cancer databases

The volume of post-genomic data has resulted in the creation of a plethora of resources for cancer research community and lead to innovative approaches to cancer prevention [16]. We summarized major sites where these data sets can be assessed in Table 1. Note that the contents of the databases are not exclusive for a specific molecular type and are partly redundant.

**Table 1:** Public databases for cancer genomics data

Resource	Description	URL	Expr	CNV	Mut	Epi	Integ	Others
The Cancer Genome Atlas (TCGA)	Copy number, gene and microRNA expression, promoter methylation, genetic alterations association with brain, lung and ovarian cancer	http://cancergenome.nih.gov/dataportal	✓	✓	✓	✓		
The International Cancer Genome Consortium (ICGC)	Full range of somatic mutations in 50 different cancer type [17]	http://dcc.icgc.org	✓	✓	✓			
NCBI dbGaP	Store individual-level phenotype, exposure, genotype and sequence data and the associations between them [18]	http://www.ncbi.nlm.nih.gov/gap/			✓			
COSMIC	Provide mutation range and frequency statistics based upon a choice of gene and/or cancer phenotype [19]	http://www.sanger.ac.uk/cosmic		✓	✓			
Oncomine	Collect gene expression, pathways, networks [20]	http://www.oncomine.org	✓					✓
Cancer Gene Census	Annotation of muted genes [21]	http://www.sanger.ac.uk/genetics/CGP/Census			✓			
Cancer Genome Anatomy Project (CGAP)	Resource of gene expression profiles of normal, pre-cancer, and cancer cells [22]	http://cgap.nci.nih.gov			✓			
Cancer Molecular Analysis Project (CMAP)	Available for analysis gene associated with oncogenesis and cancer profiles, clinical trials and therapies [23]	http://cmap.nci.nih.gov/					✓	
Cancer Biomedical Informatics Grid (caBIG)	Open access for large multi-disciplinary data sets, analysis tools, and other resources [24, 25]	https://cabig.nci.nih.gov/					✓	
caArray	Accessible array data management and allow to share data across caBIG	https://array.nci.nih.gov/caarray	✓					
Cancer Genome Wide Association Scan (caGWAS)	Integrate, query, report, and analyze significant associations between genetic variations and disease, drug response or other clinical outcomes	https://cabig.nci.nih.gov/community/tools/caGWAS					✓	
Cancer Model Database (caMOD)	Provide information about animal models for human cancer to the public research community	http://cancermodels.nci.nih.gov/camod						✓
Database for copy number alterations of cancer genome from SNP array data (caSNP)	Collect of copy number alteration (CNA) from SNP arrays	http://cistrome.dfci.harvard.edu/CaSNP		✓	✓			
Database of Differentially Expressed Proteins in Human Cancers (dbDEPC)	Provide cancer proteomics data, a resource for information on protein-level expression changes, and explore protein profile differences among different cancers [26]	http://dbdepc.biosino.org/index						✓
Cancer Genetic Markers of Susceptibility (CGEMS)	Identify common inherited genetic variations associated with risk for breast and prostate cancer	http://cgems.cancer.gov			✓			
Tumorscape	Provide copy number alterations across multiple cancer types	http://www.broadinstitute.org/tumorscape		✓	✓			
UCSC Cancer Genome Browser	Visualize, integrate and analyze cancer genomics and its associated clinical data [27]	https://genome-cancer.ucsc.edu/					✓	
Gene Expression Omnibus (GEO)	Store high-throughput functional genomic data, including those that examine genome copy number variations, chromatin structure, methylation status and transcription factor binding [28]	http://www.ncbi.nlm.nih.gov/geo	✓					
Single Nucleotide Polymorphism Database (dbSNP)	dbSNP currently classifies nucleotide sequence variations with the following types of the database: (1) single-nucleotide substitutions, (2) small insertion/deletion polymorphisms, (3) invariant regions of sequence, (4) microsatellite repeats, (5) named variants, and (6) uncharacterized heterozygous assays [29]	http://www.ncbi.nlm.nih.gov/projects/SNP/			✓			
Integrative Genomics Portal (IGP) and Integrative Genomics Viewer (IGV)	The Starr Cancer Consortium developed IGP for sharing and analysis of RNAi, copy number, gene expression and sample annotation data. Also, they provide IGV, which is a high performance desktop application that supports integrated visualization of a wide range of genomic data types including aligned sequence reads, mutations, copy number, RNAi screens, gene expression, methylation, and genomic annotations [30]	http://www.broadinstitute.org/IGP/home http://www.broadinstitute.org/igv/					✓	

Publicly available cancer databases contain gene/microRNA expression data (Expr), copy number of variations (CNV), mutations (Mut), epigenetic profiling (Epi), integration analysis (Integ), and other data (i.e., proteomics, networks, mouse models)

Cancer genomic data sources can be divided as follows:

- *Databases harboring gene/microRNA expression profiles* The discovery of gene/microRNA expression patterns provides better predictions of clinical outcome than traditional clinicopathologic standards [31] and can be used for molecular classification of human cancer [32, 33].
- *Databases for copy number of variations (CNV)* Results generated using various reliable platforms including NSG for high-resolution detection of DNA copy number changes are available [31, 34, 35]. The publicly available data generated with diverse platforms are given in the second column.
- *DNA mutation detection databases* All cancers arise as a result of the acquisition of a series of fixed DNA sequence abnormalities. These abnormalities include base substitutions, deletions, amplifications, and rearrangements [36]. Thus, the strongest predictors of risk of developing cancer and of response to therapy appear to be at the DNA level [31]. Databases were designed to store, manage, organize, and present the information on somatic mutations in cancer (i.e., COSMIC, caSNP, dbSNP). For example, COSMIC database describes somatic mutations information relating to human cancers. Recently, genome-wide somatic mutation content of tumor samples, including structural rearrangements and non-coding variants, has been included. COSMIC is now integrating this information into the database, providing full coding and genomic variant annotations for samples, both from CGP laboratories and recent publications [19].
- *Epigenetic profiles databases* The datasets include histone acetylation, histone methylation, and DNA methylation. These modifications are now thought to play important roles in the onset and progression of cancer in numerous tumor types [37].
- *Databases with integrative analyses* These databases provide results representing analysis of data across a cohort of samples where statistical methodologies and computational algorithms were applied to identify molecular subtypes from various data sources [38]. For example, the Cancer Biomedical Informatics Grid (caBIG) aims to provide a common informatics platform to the cancer research community by integrating heterogeneous datasets and the provision of open access interoperable tools (i.e., caArray, caGWAS) [16].
- *Databases with other data types* Finally, there are databases with other types of data (i.e., mouse models, phenotypic data, networks, proteomics) also aiming at collecting and providing insights into the mechanism of cancer development [38]. For example, Cancer Model Database (caMOD) provides information about animal models for human cancer [39] to the research community.

### Epitope databases

**Fig. 2:**
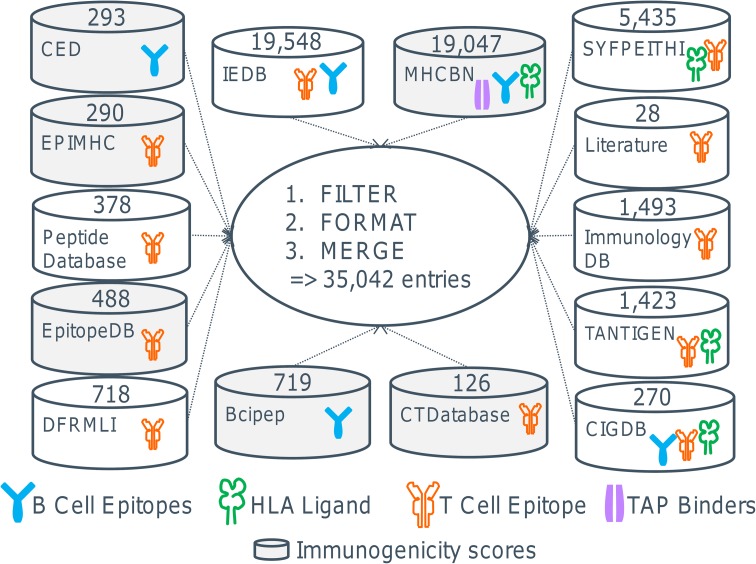
Databases for epitopes and calculation of the total number of epitopes. Shown are available databases and the number of entries in each database (see text for abbreviations). Since there is a considerable overlap between the databases, we have analyzed the data and as of to date identified the number of unique peptide sequences to be around 35,000. The number of entries per database refers only to human peptide sources

There are a number of publicly available databases containing experimentally and computationally derived information on T cell and B cell epitopes, binders to the major histocompatibility complex (MHC) molecules, and the transporter associated with antigen processing (TAP) (Table 2). Since there is a considerable overlap between the databases, we calculated the unique entries by filtering, formatting, and merging the contents of the databases. This analysis shows that there are currently about 35,000 entries for human peptides (Fig. 2).

**Table 2:** Databases containing immunogenic and non-immunogenic peptides in human

Database	Content	# Entries	URL	Reference
Bcipep	Linear B cell epitopes with descriptive immunogenicity measure	719	http://bioinformatics.uams.edu/mirror/bcipep	[40]
CED	Conformational B cell epitopes with immunoproperty description	293	http://immunet.cn/ced	[41]
CIG-DB	Publicly available epitopes that interact with IG (linear and conformational) and TCR	270	http://scchr-cigdb.jp	[42]
CTDatabase	Cancer-Testis antigens and corresponding mRNA and protein expression, and immune response	126	http://www.cta.lncc.br	[43]
DFRMLI	HLA binding peptides packed up into ready-to-train-and-test data sets, and T cell epitopes	718 TAAs	http://bio.dfci.harvard.edu/DFRMLI	[44]
EPIMHC	HLA ligands associated with high, low, moderate, or unknown binding level and a flag indicating immunogenic epitopes	290 TAAs	http://imed.med.ucm.es/epimhc	[45]
IEDB	Linear and conformational antibody and T cell epitopes cross-referenced with publications, MHC binding experiments and T cell assays	598 Conf. 18950 Lin.	http://immuneepitope.org	[46]
Immunology DB	HIV antibody epitopes (mainly from non-human sources), HIV CTL and T helper epitopes, epitope variants and escape mutations (EVEM)	1,493 T cell epitopes 2516 EVEM	http://hiv.lanl.gov/content/immunology	
MHCBN	Class I and II MHC and TAP binders associated with binding affinity and T cell activity measures, as well as non-binders	645 TAP 18,404 MHC	http://imtech.res.in/raghava/mhcbn	[47]
PeptideDatabase	T cell-defined tumor antigens	378	http://cancerimmunity.org/peptide	[48]
SYFPEITHI	MHC Class I and II binding peptides and corresponding binding motifs	5,435	http://www.syfpeithi.de	[49]
TANTIGEN	Human tumor-associated HLA ligands and T cell epitopes with detailed description for the source antigen	1,423	http://cvc.dfci.harvard.edu/tadb

Bcipep [50] and CED [41] are sources of B cell epitopes, linear and conformational, respectively. Both of them offer a descriptive measure of epitope immunoproperty. IEDB [46], MHCBN [47], and SYFPEITHI [49] are currently the largest repositories. IEDB is most frequently maintained, well annotated, and supplies broad information. It is easily queryable for tumor-related information and provides extensive experimental details. The epitope immunogenicity is quantified with affinity measures, T cell activity, or antibody binding assays. It is generated from automatically compiled publications that describe epitopes, which are classified using machine learning methods, and subsequently manually curated by senior immunologists. However, since cancer is not one of the priority diseases, for this database, cancer-related literature is not yet comprehensively covered. Thus, despite IEDB’s large size, the contents of other databases are complementary.

Unlike IEDB, MHCBN also contains information on TAP binders, in addition to peptides binding to MHC molecules. Moreover, not only the positive examples of binding proteins are collected from the literature and the available databases, but also non-binding peptides are included. It is a rich source of information, where the immunogenicity of the peptides is quantified with categorical measures (low, medium and high) of binding affinity and T cell activity; nevertheless, there is still space for improvement, for example, a more comprehensive source-protein description could alleviate interpretation. Smaller but similar to MHCBN is EPIMHC [45], also neglecting rich source-protein annotation.

SYFPEITHI has evolved from the first collection of MHC ligands into one of the largest databases. It has contributed significantly to our understanding of binding motifs and to the advances in development and validation of epitope prediction. It has been continuously maintained for more than 20 years. The constitutive MHC binders and T cell epitopes are gathered from the literature and each of them described with anchors and auxiliary anchor amino acids.

Databases developed specifically to serve for cancer vaccine target discovery are Peptide Database [48], TANTIGEN, DFRMLI [44], CIG-DB [42], and CTDatabase [43]. Peptide Database not only provides manually curated list of T cell-defined tumor antigens but also categorizes into unique, differentiation, overexpressed, and tumor specific [48]. CTDatabase presents only antigens from the last category, also referred to as Cancer-Testis. TANTIGEN follows the proposed scheme for antigen classification. Additionally, it is much more abundant and focuses on antigen annotation. It contains experimentally validated HLA ligands and T cell epitopes accompanied with the original sequence and a detailed description of the source human tumor antigens, such as multiple sequence alignment of the isoforms, gene expression profiles, database IDs in COSMIC or SwissProt for the causing substitution mutations. CIG-DB performs literature mining, training, and clustering to semi-automatically classify T cell receptors (TCR) and immunoglobulins (IG) for human and mouse into two groups: cancer therapy and hematological tumors. Additionally, it aggregates publicly available epitope sequences that interact with IG and TCR. An interesting initiative of the Dana-Farber institute is DFRMLI, a repository of immunological data sets from major public databases, intended for training and testing of machine learning methods [44].

All of the databases are populated with experimentally derived information supplied in the literature, with the exception of MHCBN and EPIMHC, which include information from available databases. There has been one attempt for computational derivation of T cell epitopes, catalogued in the HPtaa [51] database; however, it is currently not maintained and its access is impeded.

## Bioinformatics tools for cancer immunology and immunotherapy

The management and analysis of data generated with “standard” technologies like microarrays including SNP arrays and array CGH arrays has been subject of previous reviews [52–56]. In this paper, we therefore highlight NGS data analysis, since this methodology is gaining increasing popularity. Moreover, whole-genome or whole-exome sequencing provides also information of single-nucleotide variants, which can be further used to predict epitopes. Epitope prediction tools were then reviewed followed by methods for integrative data analyses and network modeling.

### Next-generation sequencing

Next-generation sequencing (NGS) has emerged with a great power to provide novel and quantitative insights into the molecular machinery inside the tumor cell. In addition to expression profiling of transcripts and genes, and detection of alternative splicing, it has enabled the discovery of single-nucleotide variants (SNV), insertions, amplifications, deletions, and inter-chromosomal rearrangements in the whole genome and transcriptome. Its potential for cancer is very far from being fully exploited, having the anticipated single-cell sequencing, for example, already appearing on the horizon. Sophisticated bioinformatics methods for analysis and interpretation of tumor sequencing data are therefore of utmost importance.

The tumor is genomically unstable. Altered ploidy, tumor heterogeneity, and normal contamination are only a few of the features characterizing the tumor sequencing data that prompt the need for new and sophisticated bioinformatics approaches. For example, according to the experience of our and other labs, the different mutation rates, allelic frequencies and structural rearrangements across cancer types, subtypes, and within the tumor itself, fail to meet the assumptions underlying the statistical methods for SNV discovery in rare diseases. Therefore, most of the currently available tools for mutation detection show limited accuracy and small overlap. A step higher to RNA level brings additional challenges for detection of somatic mutations, such as post-transcriptional modifications, RNA fidelity, allele-specific expression, and expression levels ranging between extreme values. However, analyses of RNA-Seq data are complex, and we refer the readers to a recent review [57].

Whole-genome sequencing and whole-exome sequencing have proven to be valuable methods for the discovery of the genetic causes of rare and complex diseases. Although cheaper than Sanger sequencing, whole-genome sequencing remains expensive on a grand scale. Over and above, one sequencing run provides enormous amount of data and poses considerable challenges for the analysis and interpretation. In contrast, whole-exome sequencing becomes a popular approach to bridge the gap between genome-wide comprehensiveness and cost-control by capturing and sequencing approximately 1 % of the human genome that codes for protein sequences.

The complete whole-genome or whole-exome sequence data analysis process is complex, includes multiple processing steps, is dependent on a multitude of programs and databases, and involves dealing with large amounts of heterogeneous data. Currently, there are 168 individual tools addressing some of the required analysis steps, 13 complete pipelines, and 11 workflow systems. Combining different tools and methods for analysis to obtain biological meaningful results presents a challenge. These problems can be eased by using comprehensive and intuitive pipelines that consist of combination of software tools, which are capable of analyzing all steps starting from raw sequences to a set of final annotations.

However, not all pipelines cover essential steps of read alignment, variant detection, and variant annotation. We therefore describe only the pipelines covering the entire analysis workflow: HugeSeq [58], Treat [59], and SIMPLEX [60]. HugeSeq is a fully integrated pipeline for NGS analysis from aligning reads to the identification and annotation of all types of variants (SNPs, Indels, CNVs, SVs). It consists of three main parts: (1) preparing and aligning reads, (2) combining and sorting reads for parallel processing of variant calling, and (3) variant calling and annotating. Treat is a pipeline where the user can use each of the three modules (alignment, variant calling, and variant annotation) separately or as an integrated version for an end-to-end analysis. It provides a rich set of annotations, html summary report, and variant reports in Excel format. SIMPLEX [60] is an autonomous analysis pipeline for the analysis of NGS exome data, covering the workflow from sequence alignment to SNP/DIP identification and variant annotation. It supports input from various sequencing platforms and exposes all available parameters for customized usage. It outputs summary reports and annotates detected variants with additional information for discrimination of silent mutations from variants that are potentially causing diseases.

In contrast to the pipelines described above, workflow management systems are specifically designed to compose and execute a series of data manipulation or analysis steps. Most existing systems provide graphical user interfaces allowing the user to build and modify complex workflows with little or no programming expertise. Galaxy [61] is a web-based platform where the user can perform, reproduce, and share complete analyses. Pipelines are represented as a history of user actions, which can be stored as a dedicated workflow. It contains over a hundred analysis tools and users can add new tools and share entire analysis steps and pipelines. The Taverna [62] workflow management system stores workflows in a format that is simple to share and manipulate outside the editor. Initially, it did not ship with any prepackaged NGS analysis tools and integrating tools requires some programming experience. LONI [63] is a workflow processing application that can be used to wrap any executable for use in the environment. In order to access the tools, users need to connect to either public or private pipeline servers.

### Epitope prediction tools

Point mutations, chromosomal rearrangements, translation from cryptic start sites or alternative reading frames, splicing aberrations, and over-expression have all been reported as non-conventional sources of antigens [64, 65]. Regardless of whether these genetic changes contribute to oncogenesis or not, they could affect the immune response. For the first time, comprehensive characterization of the tumor genotype is enabled by sophisticated computational analysis of deep-sequencing data. The mutational signatures can further be screened for potential impact on immune activity, in order to detect vaccine target candidates or to predict response to therapy.

Somatic amino acid substitutions and short DNA deletions and insertions that reside in exons result with changes in the protein sequences that could eventually be discriminated as non-self and potentially trigger anti-tumor behavior. Mutations could be a source of novel peptides that are presented on the cell surface by MHC molecules, where they can be recognized by T helper or cytotoxic T lymphocytes (CTL). To obtain a set of potentially immunogenic peptides, sequence windows spanning each newly introduced amino acid should be extracted, with window sizes incremented within the known epitope length range. These sequence fragments are then analyzed by epitope prediction tools. An alternative method is based on antigen–antibody interactions which play an important role in human immune response. In case when conformational epitopes are sought, the whole mutated antigen sequence is analyzed, as opposed to sequence windows, since potential structural changes should also be considered.

Epitope prediction has been a subject of study for many years, and it remains an active area of research. Many new methods have been published, and the existing tools have been considerably improved. The growth of experimental data has enabled the use of more sophisticated methods, resulting in increased prediction accuracy. Furthermore, the diversity of MHC molecules that can be studied has also increased. Binding predictions are now available for hundreds of MHC alleles, resulting in the coverage of the majority of the population. There is a plenty of reviews describing the technical background of the prediction algorithms [66–68]. Here, we describe freely available, state-of-art tools that currently stand out in the huge repertoire of methods.

#### T cell epitope prediction

The initial attempts for epitope prediction aimed at estimation of MHC binding affinity, for the purpose of reducing the list of candidate T cell epitopes. Since then, much of the efforts have been invested into MHC binding prediction. It starts with the binding motifs [49], when experimentally confirmed binders are used to create a matrix, where each element represents a score for one amino acid at a given position. The highest score is assigned to amino acids that frequently reside at the anchor position. The scores decrease reversely to frequency of occurrence of the residue down to the minimum score for amino acids that are unfavorable for binding. Later, it was confirmed that MHC binding is the best indicator of immunogenicity, and therefore, the first prediction methods are still popular. The matrix-based methods: SYFPEITHI [49] for MHC class I and II binding prediction, and BIMAS [69], intended for identification of HLA-class I binders, are widely used, particularly for prediction of HLA-A*0201 restricted epitopes [70–73]. Being one of the most frequent HLA-class I allele, HLA-A*0201 has been the first and the most widely studied. The peptides that should be selected are the 2 % of the highest scoring predictions, because they are expected to contain naturally presented T cell epitopes [69, 74], in more than 80 % of the cases for SYFPEITHI [74].

This approach assumes that each amino acid at a particular position contributes to the MHC-peptide complex stability independently of the other amino acids, which is considered as its main limitation. The growth of experimental data enabled the use of elaborated machine learning methods that capture the patterns of amino acid dependencies in the sequence. Among the matrix-based tools, stabilized matrix method (SMM) [75] and NetMHC [76] stand out for their performance [77, 78] and have been continuously upgraded. The outcome of the higher-order methods depends on the training set, for example the range of peptide lengths they output is limited to the peptide lengths used for training, which is small for long MHC class II peptides. However, given an appropriate training datasets, the higher-order methods are also more accurate.

The binding strength to the MHC class I molecules has been proved to be the most restrictive step for immunogenicity prediction and to be the easiest to estimate from the peptide sequence. However, the remaining components in the antigen presenting pathway can be used to increase the prediction confidence. There are tools that predict MHC class I pathway events, such as proteasomal cleavage and TAP transport efficiency. TAP binding should be considered with caution, because it might not be the best choice for HLA-A2 binder prediction since around 10 % of the HLA-A2 restricted peptides are transported to the endoplasmic reticulum independently of TAP. The proteasomal cleavage tools predict potential cleavage sites or most probable peptide fragments. Standalone tools for proteasomal cleavage and TAP transport did not reach as widespread acceptance as MHC prediction tools, because these events are more complicated to model and alternative pathways also interfere. In spite of that, they have contributed to greater prediction power when integrated with MHC binding predictors [79].

The tools for MHC class II binding exhibit declined performance, owing to the variable length of the peptides that bind to the open groove of the MHC class II molecule. As mentioned above, SYFPEITHI can be used for MHC class II prediction. However, it is only limited to peptides with length of 8–11 and 15 and offers small allele coverage. Tools that overcome these limitations and exhibit relatively high accuracy are netMHCIIpan [80] and TEPITOPEpan [81]. TEPITOPEpan is the predecessor of a recent upgrade of the once-most-popular tool for MHC Class II binding prediction, TEPITOPE. It is able to detect only HLA-DR binders, more than 700 allele types, shows comparable accuracy to NetMHCIIpan, and performs well in predicting binding cores.

SYFPEITHI, BIMAS, and IEDB AR occur in the majority of published papers. Even though there are more refined methods claiming higher accuracy, SYFPEITHI and BIMAS remain to be widely used. The explanation could be that they have shown good performance on HLA-A2 restricted peptides, and HLA-A2 is the most abundant, and hence, the most studied human serotype. Pan-specific methods represent state of the art [80–82]. Lack or scarcity of experimental binding information for HLA alleles, for which the sequence is known, is not a limitation anymore. This is achieved by using the peptide sequence and the contact information for the corresponding MHC molecule to train the algorithm. In this way, the algorithm is able to recognize binding potential to uncharacterized MHC molecules. Benchmark studies have estimated NetMHCpan as the most accurate pan-specific MHC binding predictor [83] and NetCTLpan as the best performing integrated approach [82].

#### B cell epitope prediction

The predictive performance of B cell epitope prediction methods has only gradually advanced over the years [84]. BepiPred predicts linear B cell epitopes by combining a hidden Markov model and two propensity scores: Levitt’s secondary structure and Parker’s hydrophilicity, achieving an AUC of 0.6 [85]. ABCPred [86] is another linear B cell predictor that achieves accuracy of ~66 % in the best case by using recurrent artificial neural networks. Choosing an epitope selection threshold for these methods requires a trade-off between sensitivity and specificity.

Most of the tools for prediction of conformational B cell epitopes require the protein structure of the antigen. Normally, the structure of the novel protein sequence resulting from genetic alterations in the tumor is not known. In such cases, sequence-based methods and auxiliary tools for structure prediction are convenient. CBTope [87] is a Support Vector Machine model trained on experimentally verified protein chains to detect antibody interacting residues. Thus, it requires only the antigen sequence as input. It reports a very high maximum accuracy of more than 85 % (AUC 0.9). The biggest drawback of CBTope is that it does not discriminate the epitope coordinates from the antigen. ElliPro [88] is more convenient method for this purpose. It generates a list of predicted linear and conformational epitopes. It was shown that the method overperforms 6 other structure-based methods with an AUC of 0.732 [88]. In case of a missing protein structure, the tool accepts protein sequence as input, which is then compared with structural templates in PDB using BLAST. A user-defined number of best-hit structural templates are used to model a 3D structure of the submitted sequence by MODELLER [89]. It identifies the components of the conformational B cell epitopes as clusters of neighboring residues based on their protrusion index values.

## Integrated data analysis and network modeling

Utilizing various high-throughput technologies for characterizing the genome, epigenome, transcriptome, proteome, metabolome, and interactome enables one to comprehensively study molecular mechanisms of cancer cells and their interactions with the immune system. The real value of the disparate datasets can be truly exploited only if the data are integrated. To our experience, it is of utmost importance to first set up a local database hosting only the necessary data. Only preprocessed and normalized data are stored in a dedicated database whereas primary data are archived at separate locations including public repositories. Although it is tempting to upload and analyze all types of data in a single system, experience shows that primary data are mostly used once. This approach is even more advisable for large-scale data including microarrays, proteomics, or NGS data. However, links to the primary data need to be secured so that later re-analyses using improved tools can be guaranteed. In this context, it is noteworthy that in the majority of published studies, the analyses were based on medium-throughput data, meaning that the number of analyzed molecular species was in the range of 100–1,000 (after filtering and pre-selection). With this number of elements, the majority of the tools perform satisfactorily on a standard desktop computer.

Once the data are integrated, that is, preprocessed and deposited in a dedicated database, tools for integrative data analysis can be applied. Only then, the results of the integration of these heterogeneous datasets will provide cancer biologists with an unprecedented opportunity: to manipulate, query, and reconstruct functional molecular networks of the cells [90]. One of the most common computational approaches to delineate functional interaction networks is based on Bayes integration [91, 92] or on a statistical method for combination of *p* values from individual data sets [93]. Additionally, network and graph theory can be applied to describe and analyze the complexity of these biological systems and subsequently visualize the networks [94, 95]. For example, to reconstruct gene co-expression networks, genes (nodes) with similar global expression profiles over samples (tumor/patients) are connected, and innovative methods can be then used to identify key transcriptional regulators (ARACNe [96], MINDy [97]).

In addition to gene expression, a number of different datasets can be integrated into networks, highlighting further information otherwise hidden in the complex data sets. Especially, protein–protein interaction data provide a meaningful complementary source and can be applied to identify relevant biological effects at the network level [53, 98]. In cancer research, a number of network modeling approaches showed to be very promising [99–104]. These network approaches enable also the inclusion of clinical data from patients, which can comprise collected data during standard treatment procedures, and during clinical trials include histopathology, cancer stages and scores, prognosis (survival time, relapse time), cancer subtypes, and cancer biology parameters like ER-status for breast cancer [53].

More recently, NGS (large-scale tumor–resequencing and whole-genome exome sequencing studies) has added a new dimension to cancer research and revolutionized our ability to characterize cancers at the gene and transcript and epigenetic levels and enables identification of immunogenic tumor mutations targetable by individualized vaccines [15, 105]. A number of integrated genome analyses approaches have recently performed on several cancer types and cohorts of patients [106–117] (see in particular The Cancer Genome Atlas (TCGA)). Using these resulting human genome data sets in conjunction with bioinformatics tools, it is possible to predict biological meaning by searching for substantially altered pathways, missense mutations that are likely to be oncogenic, or regions of altered copy numbers [106]. For this specific purpose, recently tools were developed to address which cancer genome alterations are functionally important, what pathways are affected, or what are the mutations likely to be drivers in tumor progression (NetBox [118], DriverNet [112], MEMo [119], PARADIGM [120], CHASM [121], GISTIC [122], VarScan2 [123], CONEXIC [124]).

In summary, to gain further insight into a disease state and suggest treatment strategies integrative analysis is inevitable [125]. For example, Curtis et al. [107] presented an integrated analysis of copy number and gene expression in a discovery and validation set of primary breast tumors from 2,000 patients with long-term clinical follow-up. Their results provided a novel molecular stratification of the breast cancer population, derived from the impact of somatic copy number aberrations on the transcriptome. Similarly, Ascierto et al. [126] performed comparative analysis and validated the 5 genes signature of immune response of breast cancer in two cohorts to determine whether some patients with relapse may also show expression of the immune function genes in their tumors.

## Mathematical modeling in tumor immunology and cancer immunotherapy

Modeling has been successfully applied in physiology for many decades, but only recently the quality and the quantity of biomolecular data became available for the development of causative and predictive models. Due to their importance cancer in general, tumor immunology and cancer immunotherapy in particular have also been in the focus of theoretical investigators. For example, application of theoretical techniques and the postulation of the “two hit” hypothesis in the early 1970s led to the identification of tumor-suppressor genes [127]. Later, in a landmark paper, it was shown that cancer results from evolutionary processes occurring within the body [128]. The theoretical field of cancer immunology and immunotherapy experienced similar development as the experimental: enthusiasm phase in the 1970s and 1980s, skepticism phase from mid-1980s to the end of last century, and recent renaissance phase. The availability of genomic and other types of quantitative data has recently driven the development and application of a number of mathematical models of both types, descriptive and mechanistic. In this review, we are focusing on two areas in which mathematical modeling has seen recent great progress: (a) modeling clonal evolution in cancer, and (b) modeling tumor-immune cell interaction.

### Modeling clonal evolution in cancer

Cancer progression is an evolutionary process [97] that results from accumulation of genetic and epigenetic variations in a single somatic cell. These variations are heritable and can provide the cell with a fitness advantage. The genetic changes produce phenotypic changes associated with increased proliferation capabilities, decreased death, enhanced migration and invasion, evasion of the immune system, or the ability to induce angiogenesis. Cells with advantageous mutations eventually outgrow competing cells and tumor development proceeds by successive clonal expansions. In each clonal expansion, additional mutations are accumulated that drive cancer progression and lead to more invasive phenotypes. New mutations cause the simultaneous presence of multiple subclones of cells at different malignancy levels, all sharing a common ancestor, which leads to tumor heterogeneity [129].

Because of its importance, the dynamics of the clonal cancer progression has been the subject of several mathematical studies [130–134]. Mathematical models may be used to address some of the important biological questions, such as understanding the mechanism of cancer initiation, progression, distinguishing driver from passenger mutations, defining the order of the genetic changes during progression, and understanding the therapeutic resistance. An in-depth review of the models has been recently published and is beyond the scope of this paper [135]. Here, we focus on recent studies with clinical implications.

The earliest approaches were models where mutations accumulate in a population of constant size, considering only one or two mutations [131, 134]. More recent studies have focused on the waiting time to cancer [136, 137], that is, the time until a critical number of driver mutations are accumulated and initiate the growth of carcinoma and have attempted to quantify the selective advantage of the driver mutations [130, 132, 133].

Beerenwinkel et al. [132] related the waiting time to the population size, mutation rate, and the advantage of the driver mutations and showed that selective advantage of mutations has the largest effect on the evolutionary dynamics of tumorigenesis. In a recent study, Bozic et al. [130] provided an equation for the proportion of expected passenger mutations versus the proportion of the drivers and estimated that driver mutations give an average fitness advantage of 0.4 %. Martens et al. [133] found that spatial structure, compared with non-structured cell populations assumed in other studies, increases the waiting time.

Additionally to the identification of the driver mutations and their selective advantage, it is also important to determine the order in which genetic events accumulate in tumors. The order can vary among tumors and even among different compartments of the same tumor and might explain important events in carcinogenesis. Early mutations are promising therapeutic targets, and late mutations are important in metastasis. Several mathematical models have been developed to define this order and explain important events in carcinogenesis [138, 139]. For example, Gerstung et al. [140] used a probabilistic graphical model and their results showed stronger evidence for temporal order on pathway level than on gene level, indicating that temporal ordering results from selective pressure acting at the pathway level [140].

Another important clinical problem in cancer research is the development of resistance to targeted therapies. Several models have been developed to explain the evolutionary dynamics of drug resistant cancer cells [141, 142]. In a recent study, Diaz et al. [143] showed that tumors became resistant to anti-EGFR antibodies as a result of emergence of resistance mutations in KRAS and other genes that were present in clonal subpopulation within the tumors before the initiation of the treatment.

The dynamics of cancer progression is determined not only by the mutations accumulating in the cells, but also by the tumor’s interactions with the microenvironment. There are several studies that use mathematical modeling to quantify the interactions of the tumor cells with the surrounding environment [144, 145]. In 2008, Gatenby et al. [146] proposed a model that identifies six microenvironmental barriers that tumor has to overcome to emerge as an invasive cancer. In another study, the authors used modeling to quantify the interactions between tumor cells and their surrounding stroma [147]. Their results showed that the evolution of invasiveness occurs by coupling proliferation and motility, as increased motility allows the cancerous cells to escape the microenvironmental restrictions that reduce their proliferation ability.

In summary, mathematical models can assist in the investigation of the clonal evolution of cancer and can give an important insight into the history of the disease. Understanding the evolutionary forces that drive carcinogenesis could lead to more effective methods for prevention and therapy. Over and above, mathematical models can predict and explain success or failure of anticancer drugs [148] and will be an important tool for the design of combination therapies and minimize drug resistance.

### Modeling of tumor–immune cell interactions

There is long history of theoretical studies and simulation techniques involving mathematical and computational approaches to study tumor progression and tumor–immune cell interaction. The used techniques include deterministic models, stochastic models, Petri nets, cellular automata, agent-based model, and hybrid approaches [149, 150]. A summary of different mathematical and computational techniques in cancer systems biology is given in a recent review paper [149–152].

One of the issues addressed using mathematical models in tumor–immune cell interaction was adoptive immunotherapy. Adoptive immunotherapy using tailored T cell infusion to treat malignancies has been proven to be effective in certain type of tumor [153–155]. However, there are still many unanswered questions for example how to generate a large number of tumor-specific T cells, how many T cells to use for therapy, and what schedule would be most effective [153]. Integrative mathematical modeling of tumor-immune system interactions and immunotherapy treatment could provide an analytical predictive framework to address such questions.

The interplay of different cytokines like IL-2 and transforming growth factors like transforming growth factor (TGF-β) is another aspect in the focus of theoretical research. There are several mathematical models that specifically incorporate the effect of the TGF-β protein on tumor development [156–159]. Recently, Wilson et al. [160] developed a mathematical model to highlight the fact that immunotherapy alone is not always effective in killing a tumor. Their studies provide an initial analytical framework for studying immunotherapy via TGF-β inhibition in combination with vaccine treatment, which help populations of immune cells to expand during initial phases of tumor presentation.

The effect of innovative new melanoma cancer therapies was investigated using models based on systems of differential equations [161]. Kirschner et al. [162] were one of the first to illustrate through mathematical modeling the dynamics between tumor cells, effector T cells, and IL-2. They explored the effects of adoptive cellular immunotherapy on the model and described in which circumstances the tumor can be eliminated. Other groups have developed and investigated the effect of IL-2. De Pillis et al. [163] proposed a sophisticated model involves tumor cells and specific and non-specific immune cells (i.e., nature killer (NK) cells) and employs chemotherapy and two types of immunotherapy (IL-2 supplementation and CD8^+^ T cell infusion) as treatment modalities. In the later version of the model, the concentrations of CD8^+^ cells and the NK cells of the model were changed. Then, it was possible to simulate the effect of endogenous IL-2 production on CD8^+^ cells and NK cells. Finally, it was shown that the potential patient-specific efficacy of immunotherapy may be dependent on experimentally determinable parameters [164].

One of basic concepts of immunotherapy is the improving of the ability of tumor-specific T lymphocytes. Kronik et al. [153] presented a new mathematical model developed for modeling cellular immunotherapy for melanoma. They found that the tumor-immune dynamics model provided minimal requirements (in terms of T cell dose and T cell functionality) depending on the tumor characteristics (tumor growth and size) for a clinical study [153].

In most mathematical models, the tumor cells interacting with the immune system were considered as homogeneous. Recently, Iwami et al. [165] implemented a model with in which the dynamics of tumor progression under immune system surveillance was investigated considering the effects of increasing mutation rates. It could be shown that there are three different thresholds depending on the rate of mutations and the number of variants. Until the first threshold is reached, the immune response suppresses all tumor variants (phase of tumor dormancy). After reaching the first threshold, some tumor cells are able to escape the immune response (phase of partial immunoescape). If the number of variants reaches the second threshold, all tumor cells escape the immune response (phase of complete immunoescape). After reaching the third and last threshold through the high number of variants, an error catastrophe occurs. In this phase, the original tumor can no longer expand the population and the original tumor cells go extinct. After the examination of different treatment strategies the model shows that combination of chemotherapy and immunotherapy is the therapy that could lead to tumor eradication and cure. To find the effective threshold of cytokine and adoptive T cell therapy is not only important to gain a broad understanding of the specific system dynamics but will also help to guide the development of combination therapies [163]. Kogan et al. [166] worked on generalized mathematical modeling for high grad malignant glioma-immune system interaction applied in untreated cases and under T cell immunotherapy. Their models described the dynamic of tumor cells, T cells, and quantities of secreted cytokines (TGF-β and IFN-γ). They also estimated a level of T cell infusion on a per-patient basis, clinical measurements, which effects tumor size. Moreover, their analysis suggested that the duration of treatment is necessary for adoptive cellular therapy.

In summary, mathematical models of tumor-immune interactions provide an analytical view of cancer systems biology in order to address specific questions about tumor-immune dynamics. In silico experimental models of cancer have the potential to allow researchers to refine their experimental programs with an aim of reducing costs and increasing research efficiency [167].

## Conclusion

This paper reviews bioinformatics methods used in a contemporary cancer immunology research and cancer immunotherapy. From the plethora of tools and methods for the analysis of biomolecular data, we reviewed selected topics which are of major importance for the field: databases, bioinformatics methods for NGS data, epitope prediction, integrative data analysis and network modeling, and mathematical models. Other topics are of similar importance, but due to the page limitations, these are not introduced. For example, digital pathology is gaining a major impact in research, teaching, and routine applications [168]. New devices for automated staining and high-resolution scanners are already in use and provide a wealth of high-content data (i.e., images with >100 Gbytes per slide). From these images, one can extract the number, the location, and type of infiltrating T cells and define an immune score, which is superior to the AJCC/UICC-TNM staging [9]. Without doubt, this and similar type of image-based information in combination with biomolecular measurements will be of great importance in future clinical practice. However, these datasets pose considerable technical challenges, which are only partially solved.

As of today, we and others strongly believe that NGS data will not only enable the identification of novel genes and pathways relevant for diagnosis and prediction of tumor progression but will also be fundamental in the near future in clinical practice. Specifically, whole-exome sequencing is increasingly being used to characterize the genomic landscape of the tumor showing a number of novel insights into the biology of the cancer and identifying novel therapeutic targets [169]. The current bottleneck in whole-exome sequencing projects is not the sequencing of the DNA itself but lies in the structured way of data management and the sophisticated computational analysis of the experimental data.

Cancer immunology research and cancer immunotherapy add an additional layer of complexity and require a specific solution. As NGS projects are delivering hundreds or even thousands of germline and somatic mutations per patient sample, automated tools are needed to process these datasets and predict putative epitopes. The accuracy of current T cell epitope predictors has reached a high level and hence enables researchers to focus on a subset of potential epitope candidates. To our experience, the overlap of the output of the prediction tools is not always identical, and we therefore recommend a consensus approach.

The ever-increasing amount of data as well as the heterogeneity and complexity of the datasets urge for intensified use of bioinformatics tools and mathematical methods. We strongly argue that only interdisciplinary teams can extract the relevant information and so generate knowledge from these datasets. Thus, wet-lab scientists should consider data management at the very beginning of the project and commit considerable resources to data management and analysis for several reasons. First, science is becoming increasingly driven by data as a source of hypotheses, and the ability to integrate and analyze heterogeneous data is crucial. Inclusion of additional data from public sources and integration with proprietary data can pinpoint novel molecular interactions. Second, specific projects require specific database solutions to manage the captured data and therefore specific adaptations and/or developments of databases are of utmost importance. And third, in our view, an approach by which biomedical questions are addressed through integrating experiments in iterative cycles with mathematical modeling, simulation, and theory will considerably contribute to the field.
